# Differential Impacts of Multimorbidity on COVID-19 Severity across the Socioeconomic Ladder in Hong Kong: A Syndemic Perspective

**DOI:** 10.3390/ijerph18158168

**Published:** 2021-08-02

**Authors:** Gary Ka-Ki Chung, Siu-Ming Chan, Yat-Hang Chan, Terry Cheuk-Fung Yip, Hon-Ming Ma, Grace Lai-Hung Wong, Roger Yat-Nork Chung, Hung Wong, Samuel Yeung-Shan Wong, Eng Kiong Yeoh, Michael Marmot, Jean Woo

**Affiliations:** 1 CUHK Institute of Health Equity, The Chinese University of Hong Kong, Hong Kong SAR, China; smcha2@cityu.edu.hk (S.-M.C.); lucachan@cuhk.edu.hk (Y.-H.C.); rychung@cuhk.edu.hk (R.Y.-N.C.); hwong@swk.cuhk.edu.hk (H.W.); yeungshanwong@cuhk.edu.hk (S.Y.-S.W.); yeoh_ek@cuhk.edu.hk (E.K.Y.); m.marmot@ucl.ac.uk (M.M.); jeanwoowong@cuhk.edu.hk (J.W.); 2 Department of Social and Behavioural Sciences, City University of Hong Kong, Kowloon Tong, Hong Kong SAR, China; 3 Department of Medicine and Therapeutics, Prince of Wales Hospital, Shatin, New Territories, Hong Kong SAR, China; terryfungyip@gmail.com (T.C.-F.Y.); mhm463@ha.org.hk (H.-M.M.); wonglaihung@gmail.com (G.L.-H.W.); 4 Medical Data Analytic Centre, The Chinese University of Hong Kong, Hong Kong SAR, China; 5 Institute of Digestive Disease, The Chinese University of Hong Kong, Hong Kong SAR, China; 6 The Jockey Club School of Public Health and Primary Care, The Chinese University of Hong Kong, Hong Kong SAR, China; 7 CUHK Institute of Ageing, The Chinese University of Hong Kong, Hong Kong SAR, China; 8 Department of Social Work, The Chinese University of Hong Kong, Hong Kong SAR, China; 9 UCL Institute of Health Equity, UCL Research Department of Epidemiology and Public Health, University College London, London WC1E 6BT, UK

**Keywords:** COVID-19, multimorbidity, socioeconomic inequalities, Hong Kong

## Abstract

The severity of COVID-19 infections could be exacerbated by the epidemic of chronic diseases and underlying inequalities in social determinants of health. Nonetheless, there is scanty evidence in regions with a relatively well-controlled outbreak. This study examined the socioeconomic patterning of COVID-19 severity and its effect modification with multimorbidity in Hong Kong. 3074 local COVID-19 cases diagnosed from 5 July to 31 October 2020 were analyzed and followed up until 30 November 2020. Data on residential addresses, socio-demographic background, COVID-19 clinical conditions, and pre-existing chronic diseases of confirmed cases were retrieved from the Centre for Health Protection and the Hospital Authority. Results showed that, despite an independent adverse impact of multimorbidity on COVID-19 severity (aOR = 2.35 [95% CI = 1.72–3.19]), it varied across the socioeconomic ladder, with no significant risk among those living in the wealthiest areas (aOR = 0.80 [0.32–2.02]). Also, no significant association of the area-level income-poverty rate with severe COVID-19 was observed. In conclusion, the socioeconomic patterning of severe COVID-19 was mild in Hong Kong. Nonetheless, socioeconomic position interacted with multimorbidity to determine COVID-19 severity with a mitigated risk among the socioeconomically advantaged. Plausible explanations include the underlying socioeconomic inequalities in chronic disease management and the equity impact of the public-private dual-track healthcare system.

## 1. Introduction

Evidence on socioeconomic inequalities in COVID-19 severity is rapidly growing. Severe COVID-19 outcomes, in terms of hospitalization, admission to intensive care unit, and deaths, are highly concentrated among the socioeconomically disadvantaged groups and communities in the world [1]. Nonetheless, the socioeconomic position does not stand alone as the sole determinant of severe COVID-19. As suggested by Bambra et al. [2], socioeconomic inequalities in COVID-19 severity and related mortality arise as a result of “a syndemic of COVID-19, socioeconomic inequalities in chronic diseases and the social determinants of health.” With the epidemiological transition over recent decades, chronic diseases have become a major public health challenge in many regions of the world. The most common chronic diseases nowadays, including cardiovascular diseases, diabetes, chronic kidney diseases, and chronic respiratory diseases, are closely linked to COVID-19 severity [3]. More importantly, as COVID-19 is increasingly recognized as a “complex multi-system clinical syndrome” [4], individuals with multiple pre-existing chronic diseases (i.e., multimorbidity) are at particularly high risk of developing severe COVID-19 if infected. In particular, the burden of multimorbidity has been strongly patterned across the socioeconomic ladder [5,6,7], which could be attributable to a range of social determinants of health, including greater barriers to engagement in self-management, lower access to a regular source of primary care, and poorer continuity and integration of care among the socioeconomically disadvantaged [7,8]. Altogether, socioeconomic disadvantage, and the corresponding exposure to health-compromising social determinants of health, as well as the devastating epidemic and socioeconomic patterning of chronic diseases, may interact with each other to exacerbate the severity of COVID-19. However, such a potential interaction has yet been examined empirically and has remained a knowledge gap in COVID-19 research to be addressed in this study.

Despite the seemingly consistent socioeconomic patterning of COVID-19 severity as reported in a recent international review on socioeconomic inequalities and COVID-19 [1], a predominantly large portion of the relevant existing studies have originated from North America and Western European countries, which have been severely struck by the COVID-19 pandemic. While there is a growing body of research from South America [9,10,11], evidence in Asian regions is extremely scarce. To the best of our knowledge, only one study in Japan specifically examined the relationship of area-level socioeconomic measures with COVID-19 mortality and revealed consistent socioeconomic patterning as observed in the Western countries [12]. Given the relatively high COVID-19 incidence rates, and hence the heavily overwhelmed healthcare system in these regions, it is no surprise that profound inequalities in COVID-19 severity were observed as the socioeconomically disadvantaged tend to have lower access to timely treatment and quality care once being infected [2,13]. However, the presence or extent of socioeconomic inequalities in COVID-19 severity can vary across regions with different magnitudes of COVID-19 spread, designs, and capacity of the healthcare system, as well as underlying social inequalities. Research focusing on regions of starkly different social contexts could therefore present a new perspective on the underlying determinants of severe COVID-19 across the socioeconomic ladder.

Hong Kong could serve as an exemplary setting for an in-depth investigation of the socioeconomic inequalities in COVID-19 severity. Compared with many other regions with widespread community COVID-19 outbreaks, Hong Kong has a relatively low COVID-19 incidence with less than 9000 confirmed cases and 150 deaths by the end of 2020 [14,15]. In addition to the better-controlled COVID-19 outbreak, the public-private dual-track healthcare system in Hong Kong also has a strong equity impact on both COVID-19 treatment and socioeconomic inequalities in chronic disease management. While the vast majority of inpatient services are provided by the public sector, which is tax-funded with low additional out-of-pocket fees at the point of care, primary care is largely provided by the private sector with over 70% market share [16]. The out-of-pocket payment for private primary care services casts a heavy burden on the socioeconomically disadvantaged, especially since they are more likely to suffer from multimorbidity [17], less likely to have private medical insurance or employer-provided medical benefits, and hence have lower access to timely and integrated primary care [18,19,20,21]. Taken together, they also tend to experience difficulty in managing their chronic diseases during the COVID-19 period [22].

To have a better understanding of the socioeconomic patterning of COVID-19 severity and its underlying determinants, this cross-sectional study aims to (i) assess the independent associations of socioeconomic position and multimorbidity with severe COVID-19, and (ii) explore the potential interaction between socioeconomic position and multimorbidity on COVID-19 severity, in the context of Hong Kong. While the fragmentation of data sources is the key barrier to most research on such an interaction effect, this study achieved data linkage across past medical records of confirmed cases and their COVID-19 clinical condition, as well as the proxy socioeconomic measures driven by their residential addresses, which enabled an empirical investigation into the syndemic nature of COVID-19 based on the context of Hong Kong.

## 2. Materials and Methods

### 2.1. Study Population and Data Source

This study was based on two data sources. First, we collected data from the Centre for Health Protection (CHP) of Hong Kong, which provides daily updates on individual laboratory-confirmed cases via its official website [14]. Data on age, sex, residency, case classification, onset date, reported date, symptoms at diagnosis, the status of hospitalized/discharged/deceased, self-reported residential address, and travel history of all confirmed cases were obtained [23]. Additional data on the clinical conditions of confirmed COVID-19 cases were obtained from the daily press releases of the Hospital Authority (HA) of Hong Kong [24]. Second, we retrieved previous medical records of confirmed COVID-19 cases over the past 20 years from the Clinical Data Analysis and Reporting System (CDARS) under the management of the HA [25]. CDARS is an electronic healthcare database that covers the patients’ demographic, death, diagnoses, procedures, drug prescription and dispensing history, and laboratory results from all public hospitals and clinics in Hong Kong which represent inpatient data of around 90% of the total Hong Kong population [26], and has been used in previous territory-wide studies on COVID-19 in Hong Kong [27,28,29]. The data retrieval from CDARS was approved by the Joint Chinese University of Hong Kong—New Territories East Cluster Clinical Research Ethics Committee.

In this study, we considered all confirmed local cases and cases epidemiologically linked with a local case (*n* = 3436) reported by the CHP from 5 July 2020 to 31 October 2020. After excluding 118 cases who cannot be identified in the CDARS, and 244 cases with missing, invalid, or multiple local residential addresses as reported by the CHP, 3074 cases were included as the study sample and followed up until 30 November 2020.

### 2.2. Measurements

#### 2.2.1. COVID-19 Severity

The severity of COVID-19 cases was determined by the daily update of the clinical conditions of confirmed cases retrieved from the HA press releases [24]. Based on a unified classification scheme on clinical conditions adopted by all the 16 public hospitals that treated COVID-19 cases, in-charge physicians would continuously monitor the clinical conditions of COVID-19 cases and classified them into four groups—(i) critical: require intubation, or extracorporeal membrane oxygenation, or in shock; (ii) serious: require oxygen supplement of three liters or more per minute; (iii) stable: with mild influenza-like illness symptoms and; (iv) satisfactory: progressing well and likely to be discharged soon [30]. In addition, death cases were reported in separate HA press releases and then summarized by the CHP [14].

In this study, COVID-19 cases who died or have ever been classified as critical or serious for one or more days during the study period were considered “severe”. The rest of cases were deemed “stable”, as the HA press releases only reported daily discharged cases without distinguishing satisfactory cases from the stable ones. Furthermore, cases who have not been classified as critical or serious but have not yet been discharged by 30 November 2020 (at least one month since diagnosis) were also considered “stable”.

#### 2.2.2. Socioeconomic Position

The self-reported residential addresses of confirmed cases were adopted to generate the area-level income-poverty rates as the proxy measures of their socioeconomic positions. First, we classified the confirmed cases into 154 large tertiary planning units (TPUs) demarcated by the Planning Department of Hong Kong [31]. Then, based on the data of the 2016 by-census from the Census and Statistics Department of Hong Kong, the median monthly household income with respect to household sizes was obtained [31]. We then estimated the number of households living with less than half of the corresponding median monthly household income by different household sizes in each TPU. By multiplying the estimated number of households by household sizes, we calculated the total number of persons living under income-poverty in each TPU and then divided this by the total population in the corresponding TPUs. Finally, we applied the resultant proportions (i.e., area-level income-poverty rates) to all cases according to the corresponding TPUs of their residential addresses. The area-level income-poverty rates in TPUs were grouped into tertiles for analysis.

#### 2.2.3. Multimorbidity

The medical history of the confirmed cases since 1 January 2000 was retrieved from CDARS. Eight broad categories of chronic diseases were identified, including (i) cardiovascular diseases, (ii) digestive diseases, (iii) diabetes, (iv) cancers, (v) nervous system diseases, (vi) respiratory diseases, (vii) kidney diseases, and (viii) human immunodeficiency virus (HIV) infections. While most chronic diseases were identified solely by their corresponding International Classification of Diseases, Ninth Revision, Clinical Modification (ICD-9-CM) diagnosis codes (Table S1), additional data on clinical measurements and drug prescriptions were used to define certain diagnoses. Specifically, hypertension was defined by any use of antihypertensive drugs and/or ICD-9-CM diagnosis codes, whereas diabetes was defined by exposure to any antidiabetic agents, and/or hemoglobin A_1c_ ≥ 6.5%, and/or fasting plasma glucose ≥ 7 mmol/L, and/or the ICD-9-CM diagnosis codes for diabetes mellitus [32]. Hence, confirmed cases who had two or more chronic disease categories prior to COVID-19 diagnosis were considered as having multimorbidity.

### 2.3. Statistical Analysis

Descriptive statistics of confirmed cases across tertiles of area-level income-poverty rate were derived. Continuous variables are presented as the mean with standard deviations (SD) and categorical variables as count numbers with percentages. Chord diagrams [33] were also used to illustrate the relative frequency of disease combinations among cases with multimorbidity across tertiles of area-level income-poverty rate. As for multivariable analysis, binary logistic regression on the associations of area-level income-poverty rate and multimorbidity with COVID-19 severity was employed, with adjustments for age, sex, presence of symptom onset, housing type (i.e., public rental housing, subsidized home ownership, private housing, residential care homes, and others including villages, industrial and commercial buildings, staff quarters), and area-level population density (i.e., the number of residents divided by land area in each large TPU obtained from the Planning Department of Hong Kong, and then re-grouped into tertiles). Multi-level modeling was not adopted in the main analysis because of negligible random intercepts across the TPUs after adjustments for age and sex (data not shown); nonetheless, a sensitivity analysis based on a multi-level binary logistic regression with random effects specified for all individual-level variables was performed to ensure the robustness of our results. To test the effect modification of multimorbidity on COVID-19 severity across tertiles of area-level income-poverty rate using binary logistic regression model, a new variable was derived with the following six groups: (i) non-multimorbid with a low income-poverty rate; (ii) multimorbid with a low income-poverty rate; (iii) non-multimorbid with a medium income-poverty rate; (iv) multimorbid with a medium income-poverty rate; (v) non-multimorbid with a high income-poverty rate and; (vi) multimorbid with a high income-poverty rate. With reference to the methods and result presentation for effect modification recommended by Knol and VanderWeele [34,35], the odds ratios of multimorbidity within the strata of area-level income-poverty rate, as well as measures of effect modification on the multiplicative scale and additive scale, in terms of relative excess risk due to interaction (RERI), were estimated with the same set of confounding control. Sensitivity analyses using the quartiles of the area-level income-poverty rate were also conducted to ensure the robustness of our results. STATA version 14 (StataCorp LLC, College Station, TX, USA) and R version 3.4.1 (R Foundation for Statistical Computing, Vienna, Austria) were employed for statistical analyses. All statistical tests were two-tailed with a significance level of *p*-value < 0.05.

## 3. Results

Among the 3074 cases with a mean age of 48.5 (median = 50.5) and an SD of 20.2 (interquartile range = 30.0), slightly less than half (48.2%) were male (Table 1). Almost one-tenth of the cases (9.1%) had severe COVID-19. Most of the cases (82.8%) were symptomatic on presentation, and more than one-fifth (22.3%) were multimorbid prior to COVID-19 diagnosis. In terms of their socioeconomic background, 12.4% of the cases lived in areas with a low income-poverty rate (i.e., the wealthiest tertile of TPUs in Hong Kong), whereas 43.4% and 42.2% lived in areas with a medium and high income-poverty rate, respectively. The distribution patterns of housing type and population density were significantly different across the area-level income-poverty rate (both *p <* 0.001).

**Figure 1:**
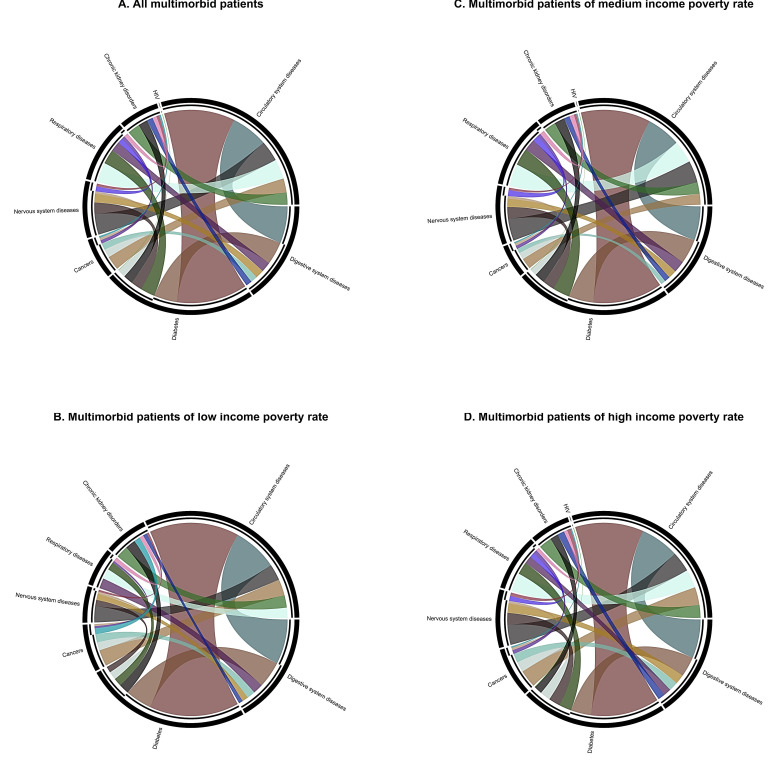
Relative frequency of disease combinations among COVID-19 cases with multimorbidity. (**A**) All multimorbid patients; (**B**) multimorbid patients of low income-poverty rate; (**C**) multimorbid patients of medium income-poverty rate; (**D**) multimorbid patients of high income-poverty rate.

In addition, the relative frequencies of chronic disease combinations among multimorbid cases across area-level income-poverty rates are displayed in Figure 1. In general, no apparent differences in the disease combinations were observed, except a slightly higher relative frequency of the combination between cardiovascular diseases and diabetes, as well as lower proportions of nervous system diseases and respiratory diseases among cases who lived in areas with a low income-poverty rate.

As shown in Table 2, being older (aOR = 1.08 [95% CI: 1.07–1.09] per year increase, *p <* 0.001), male (aOR = 2.91 [2.16–3.92], *p <* 0.001), symptomatic (aOR = 3.17 [1.98–5.06], *p <* 0.001) and multimorbid (aOR = 2.35 [1.72–3.19], *p <* 0.001) were independently associated with severe COVID-19. Nonetheless, no statistically significant associations with severe COVID-19 were observed for housing type, area-level income-poverty rate, and population density. Sensitivity analysis, based on multi-level binary logistic regression with random effects specified for all individual-level variables, showed comparable results (Table S2).

Nonetheless, the effect of multimorbidity on COVID-19 severity varied across income-poverty rates (Table 3). Among cases living in areas with a low income-poverty rate, multimorbidity did not exhibit significant association with COVID-19 severity (aOR = 0.80 [0.32–2.02], *p* = 0.636). However, significantly increased odds of multimorbidity were observed among cases who lived in areas with a medium and high income-poverty rate (aOR = 2.88 [1.86–4.45], *p <* 0.001; aOR = 2.38 [1.52–3.74], *p <* 0.001, respectively). Their corresponding measures of effect modification on both multiplicative and additive scales were statistically significant (all *p <* 0.035). Sensitivity analysis on the effect modification across quartiles of the area-level income-poverty rate showed consistent results (Table S3).

## 4. Discussion

This study is the first to assess the socioeconomic patterning of COVID-19 severity in Hong Kong, where the COVID-19 incidence is relatively low. To the best of our knowledge, this is also the world’s first empirical study to examine the potential effect modification of multimorbidity on COVID-19 severity across the socioeconomic ladder. Our findings revealed no apparent socioeconomic inequalities in COVID-19 severity. Nonetheless, socioeconomic position interacted with multimorbidity to determine the risk of COVID-19 severity. Despite the overall independent effect of multimorbidity on severe COVID-19, its adverse impact appeared to be strong among the cases of lower socioeconomic position but largely attenuated among their wealthiest counterparts.

In contrast to the profound inequalities widely observed in North America and Western European countries [1], the socioeconomic patterning of COVID-19 severity is mild in Hong Kong, which can be attributed to the relatively capacious and equitable inpatient care. With the cardinal healthcare principle of the Hong Kong Government that “no one should be denied adequate healthcare due to lack of means,” [36] more than 90% of inpatient services, in terms of hospital bed days, are provided and highly subsidized by the tax-funded public sector at the HA [16]. Given the comparatively successful COVID-19 control in Hong Kong that keeps the public sector from being seriously overwhelmed with the emerging disease, public hospitals manage to provide treatments to all confirmed COVID-19 cases who require inpatient care. The airborne isolation wards, and beds in public hospitals, have constantly been operating in their full capacity to accommodate the surge in COVID-19 cases, while the demand pressure is alleviated by the second-tier isolation wards and community isolation facilities for recovering patients in a stable condition to continue isolation and treatment [37,38]. Together with the prioritization of healthcare resources for the COVID-19 outbreak, universal access to treatment and services related to COVID-19 with similar quality of need-based inpatient care in public hospitals is upheld regardless of the socioeconomic background of the COVID-19 patients.

Despite no direct socioeconomic inequalities in COVID-19 severity, the impact of multimorbidity on severe COVID-19 differed across the socioeconomic ladder. While significant adverse effects of multimorbidity were observed among the cases living in areas with a medium and high income-poverty rate, its impact was close to null among those living in wealthier areas. As suggested by the existing literature, the independent adverse impact of multimorbidity on severe COVID-19 may operate through pre-disposing weakening of endothelial functions, pro-inflammatory profiles that provoke cytokine storm in severe COVID-19 cases, and cellular senescence especially among older adults [39,40]. Nonetheless, these possible physiological mechanisms, together with our observed similar disease combinations across the socioeconomic ladder among COVID-19 cases with multimorbidity (Figure 1), are insufficient to explain the mitigated risk of multimorbidity on COVID-19 severity among cases of higher socioeconomic position.

As mentioned above, primary care in Hong Kong is primarily provided by the private sector, resulting in pro-rich inequalities in outpatient visits [16]. Patients of a lower socioeconomic position rely heavily on the almost free but limited public outpatient services with a long waiting time, whereas those of a higher socioeconomic position can afford fast-track access and regular sources of private primary care in addition to public services [18,19,20,21]. As reported by a previous local study, patients who receive primary care primarily from private general practitioners reported better primary care experiences compared with those receiving care primarily from public clinics, which was attributable to the higher accessibility (among those who can afford it) and better person-focused care in the private sector [18]. The persistent underlying disparity in access to primary care is further exacerbated during the COVID-19 period, as non-emergency and non-essential services in public outpatient clinics have been greatly reduced to focus the constrained healthcare resources and manpower on combating COVID-19 [41]. Together with the impact of social distancing policies, it is common that patients with multimorbidity substantially default medical appointments for fear of contracting COVID-19 from clinics and hospitals [42]. Thus, it is more difficult to manage chronic diseases among the socioeconomically disadvantaged [22]. Although the advancement of telemedicine and digital health innovations may partially alleviate the challenge of healthcare delivery due to COVID-19, it is likely to have unintended impacts on health equity as those of a lower socioeconomic position tend to benefit less from these solutions [43]. In addition to access to primary care, the differential quality, continuity, and integration of primary care across the socioeconomic ladder [18,19] could have also contributed to the observed socioeconomic difference in the risk of multimorbidity on COVID-19 severity. The lack of integrated care, commonly experienced by the socioeconomically disadvantaged with pre-existing multimorbidity, is likely to harm the disease prognosis of COVID-19 because of uncoordinated multiple treatments and over-medication [44,45,46]. In summary, the better access to integrated primary care among patients of higher socioeconomic position even during the COVID-19 period, coupled with their generally better health literacy, health information-seeking behaviors, and treatment compliance [47], may have enabled them to achieve more effective chronic disease management and therefore be better protected from the excess risk of severe COVID-19 due to multimorbidity if becoming infected. Altogether, to achieve an equitable and sustainable recovery from COVID-19 and better preparedness for the next potential worldwide catastrophe, policymakers should focus on chronic disease prevention and capacity building of the healthcare system through an equity lens, and meanwhile target the wider social determinants of health to mitigate the long-standing social inequalities in societies.

The strengths of our study include the use of a territory-wide cohort that covers about 90% of the inpatient services and essentially all the COVID-19 cases in Hong Kong up to the end of our study period. The data linkage between clinical data and socioeconomic indicators of confirmed local COVID-19 cases also enabled an in-depth understanding of the effect modification of multimorbidity on COVID-19 severity across the socioeconomic ladder. However, our study also has several caveats. First, since the CHP released limited information on the confirmed COVID-19 cases, we relied on patient-reported residential addresses to determine the area-level income-poverty rates of their corresponding TPUs as a proxy measure of socioeconomic position. While the use of the area-level income-poverty rate, rather than housing type, allows meaningful comparison with relevant overseas studies using regional income or poverty rate as the key socioeconomic indicators [1], it may overlook other dimensions of socioeconomic position such as education, occupation, and deprivation [48,49,50,51]. Residual confounding is also possible due to data unavailability. For example, the potential effect of spatial accessibility to public clinics [52] was not considered; nonetheless, no significant difference in days from symptom onset to diagnosis was observed across the socioeconomic ladder in our sample (data not shown). Second, we missed 118 out of 3436 (3.4%) of local COVID-19 cases reported by the CHP during the study period because of unsuccessful identification in the CDARS. Another 244 cases (7.1%) with missing, invalid, or multiple residential addresses were also excluded. Therefore, our results may be subject to selection bias, albeit minimal. Third, we focused on locally confirmed cases diagnosed between 5 July and 31 October 2020, but not those diagnosed in the earlier waves because the HA press releases only started providing daily updates on the clinical conditions of COVID-19 cases after 11 May 2020, and there had been no major outbreaks in Hong Kong until 5 July 2020. Therefore, our observed mild socioeconomic patterning of COVID-19 severity may be partly attributable to the strengthened preparedness and surveillance measures adopted by the Hong Kong Government in the later phase of the local outbreak. Last, inaccurate entry of diagnosis codes of chronic diseases may affect the reliability of this study; nonetheless, such an ascertainment bias was minimized by including data on laboratory measurements, drug prescriptions, and dispensing history for certain diagnoses (i.e., hypertension and diabetes).

## 5. Conclusions

Socioeconomic inequalities in severe COVID-19 are largely avoidable. The case in Hong Kong suggests that a better COVID-19 containment and a relatively equitable inpatient care, which treated all the infected cases in need irrespective of their income level and multimorbidity status, could provide a safety net to the socioeconomically disadvantaged and vulnerable groups against emergency and severe COVID-19 conditions, as reflected by the observed mild socioeconomic patterning of COVID-19 severity in Hong Kong compared with the Western countries. However, the COVID-19 outbreak also exposed underlying fault lines in Hong Kong. The differential risks of severe COVID-19 among multimorbid cases across the socioeconomic ladder imply the pervasiveness of deeply entrenched socioeconomic inequalities in multimorbidity and access to primary care for chronic disease management, which have been further amplified due to the COVID-19 outbreak.

## Figures and Tables

**Table 1:** Basic characteristics of local COVID-19 cases with valid residential addresses diagnosed between 5 July and 31 October 2020 in Hong Kong (*n* = 3074).

		Total Sample	Area-Level Income-Poverty Rate			
			Low (*n* = 381, 12.4%)	Medium (*n* = 1335, 43.4%)	High (*n* = 1358, 42.2%)	
		*n* (%) or Mean (±SD)	*n* (%) or Mean (±SD)	*n* (%) or Mean (±SD)	*n* (%) or Mean (±SD)	*p*-value ^a^
Age, year		48.5 (±20.2)	45.4 (±20.1)	50.8 (±20.4)	47.1 (±19.8)	0.347
Sex						0.468
	Female	1593 (51.8)	199 (52.2)	675 (50.6)	719 (52.9)	
	Male	1481 (48.2)	182 (47.8)	660 (49.4)	639 (47.1)	
COVID-19 disease status						0.735
	Stable	2793 (90.9)	357 (93.7)	1195 (89.5)	1241 (91.4)	
	Severe	281 (9.1)	24 (6.3)	140 (10.5)	117 (8.6)	
Presence of symptom onset						0.070
	Asymptomatic	529 (17.2)	52 (13.6)	231 (17.3)	246 (18.1)	
	Symptomatic	2545 (82.8)	329 (86.4)	1104 (82.7)	1112 (81.9)	
Multimorbidity						0.511
	Non-multimorbid	2387 (77.7)	312 (81.9)	994 (74.5)	1081 (79.6)	
	Multimorbid	687 (22.3)	69 (18.1)	341 (25.5)	277 (20.4)	
Housing type						<0.001
	Public rental housing	1342 (43.7)	46 (12.1)	621 (46.5)	675 (49.7)	
	Subsidized home ownership	358 (11.6)	29 (7.6)	205 (15.4)	124 (9.1)	
	Private housing	1087 (35.4)	251 (65.9)	391 (29.3)	445 (32.8)	
	Residential care homes	109 (3.5)	3 (0.8)	70 (5.2)	36 (2.7)	
	Others	178 (5.8)	52 (13.6)	48 (3.6)	78 (5.7)	
Area-level population density						<0.001
	Low	474 (15.4)	194 (50.9)	129 (9.7)	151 (11.1)	
	Medium	1065 (34.6)	77 (20.2)	459 (34.4)	529 (39.0)	
	High	1535 (49.9)	110 (28.9)	747 (56.0)	678 (49.9)	

^a^ Mantel–Haenszel test for trend was used for categorical variables while simple linear regression was used for continuous variables.

**Table 2:** Associations of socioeconomic position, multimorbidity, and other risk factors with COVID-19 severity.

		Severe COVID-19	
		aOR [95% CI] ^a^	*p*-value
Age, year		1.08 [1.07–1.09]	<0.001
Sex			
	Female	Ref	
	Male	2.91 [2.16–3.92]	<0.001
Presence of symptom onset			
	Asymptomatic	Ref	
	Symptomatic	3.17 [1.98–5.06]	<0.001
Multimorbidity			
	Non–multimorbid	Ref	
	Multimorbid	2.35 [1.72–3.19]	<0.001
Housing type			
	Public rental housing	Ref	
	Subsidized home ownership	1.03 [0.64–1.66]	0.917
	Private housing	1.22 [0.86–1.73]	0.256
	Residential care homes	1.12 [0.66–1.92]	0.666
	Others	2.00 [0.96–4.15]	0.064
Area-level income-poverty rate			
	Low	Ref	
	Medium	1.28 [0.74–2.23]	0.375
	High	1.40 [0.81–2.43]	0.224
Area-level population density			
	Low	Ref	
	Medium	1.58 [0.90–2.77]	0.112
	High	1.56 [0.89–2.72]	0.120

^a^ Variables listed above were mutually adjusted in the binary logistic regression model.

**Table 3:** Modification of the effect of multimorbidity on COVID-19 severity by tertiles of area-level income-poverty rate.

		Non-Multimorbid		Multimorbid		Effect for Multimorbidity within Strata of Area-Level Income-Poverty Rate	
		aOR [95% CI] ^a^	*p*-value	aOR [95% CI] ^a^	*p*-value	aOR [95% CI] ^a^	*p*-value
Area-level income-poverty rate							
	Low	Ref		0.80 [0.32–2.02]	0.636	0.80 [0.32–2.02]	0.636
	Medium	0.69 [0.35–1.38]	0.293	1.98 [1.00–3.92]	0.049	2.88 [1.86–4.45]	<0.001
	High	0.85 [0.43–1.66]	0.627	2.01 [1.02–3.99]	0.045	2.38 [1.52–3.74]	<0.001
Measure of effect modification of multimorbid * medium income-poverty rate on multiplicative scale: ratio of aORs [95% CI] = 3.60 [1.31–9.91]; *p*-value = 0.013. Measure of effect modification of multimorbid * high income-poverty rate on multiplicative scale: ratio of aORs [95% CI] = 2.98 [1.08–8.23]; *p*-value = 0.035. Measure of effect modification of multimorbid * medium income-poverty rate on additive scale: RERIOR [95% CI] = 1.62 [0.63–2.62]; *p*-value = 0.001. Measure of effect modification of multimorbid * high income-poverty rate on additive scale: RERIOR [95% CI] = 1.28 [0.31–2.26]; *p*-value = 0.010.							

^a^ Adjusted for age, sex, presence of symptom onset, housing type, and area-level population density.

## Data Availability

Data on residential addresses, socio-demographic background, and clinical conditions of confirmed cases were obtained from the Centre for Health Protection, the Planning Department, and the Census and Statistics Department of the Hong Kong Government, which are all open to the public on their official websites. Data from the local online platform “covid19.vote4.hk—COVID-19 in HK”, which integrated information reported by the Centre for Health Protection, are also freely accessible at https://wars.vote4.hk/en (accessed on 22 February 2021). However, data on patient’s clinical history were obtained from a third party and would not be publicly available. Deidentified patient data were collected from the Clinical Data Analysis and Reporting System (CDARS) under the management of Hospital Authority, Hong Kong. All patients’ data were deidentified in CDARS to ensure confidentiality.

## Supplementary Materials

The following are available online at https://www.mdpi.com/article/10.3390/ijerph18158168/s1, Table S1: International Classification of Diseases, Ninth Revision, Clinical Modification (ICD-9-CM) diagnosis and procedure codes for chronic diseases, Table S2: Associations of socioeconomic position, multimorbidity and other risk factors with COVID-19 severity based on multi-level binary logistic regression with random effects specified for all individual-level variables, Table S3: Modification of the effect of multimorbidity on COVID-19 severity by quartiles of area-level income-poverty rate.
