# Supplementary material for: Differential Impacts of Multimorbidity on COVID-19 Severity across the Socioeconomic Ladder in Hong Kong: A Syndemic Perspective
Source: Int J Environ Res Public Health. 2021 Aug 2;18(15):8168. doi: 10.3390/ijerph18158168 (PMC8346110; doi:10.3390/ijerph18158168)
Supplement: Supplementary file 1 [file ijerph-18-08168-s001.zip › ijerph-1273590-supplementary.pdf]

Table S1. International Classification of Diseases, Ninth Revision, Clinical Modification (ICD-9-CM) diagnosis and procedure codes for chronic diseases.

Chronic diseases	ICD-9-CM Code	Description
**Cardiovascular diseases**		
Hypertension and hypertensive diseases	401	Essential hypertension
	402	Hypertensive heart disease
	403	Hypertensive chronic kidney disease
	404	Hypertensive heart and chronic kidney disease
	405	Secondary hypertension
Ischemic heart disease	410	Acute myocardial infarction
	411	Other acute and subacute forms of ischemic heart disease
	412	Old myocardial infarction
	413	Angina pectoris
	414	Other forms of chronic ischemic heart disease
Cardiac dysrhythmias	427	Cardiac dysrhythmias
Heart failure	428	Heart failure
**Digestive diseases**		
Peptic ulcer	530.2	Ulcer of esophagus
	531	Gastric ulcer
	532	Duodenal ulcer
	533	Peptic ulcer site unspecified
	534	Gastrojejunal ulcer
Chronic liver disease, liver failure, liver cirrhosis and complications	070.2-3	Chronic hepatitis B
	070.41, 44	Hepatitis C with hepatic coma
	070.51, 54	Hepatitis C without mention of hepatic coma
	V02.61	Hepatitis B carrier
	V02.62	Hepatitis C carrier
	070.42, 52	Hepatitis delta without mention of active hepatitis B
	275.0	Hemochromatosis
	275.1	Wilson's disease
	273.4	Alpha-1 antitrypsin disease
	570	Acute and subacute necrosis of liver
	571	Chronic liver disease and cirrhosis
	572	Liver abscess and sequelae of chronic liver disease
	573.0-5	Other disorders of liver
	348.3	Encephalopathy, unspecified
	349.82	Toxic encephalopathy
	456.0, 20	Esophageal varices with bleeding
	456.1, 21	Esophageal varices without bleeding
	456.8:1-2	Bleeding gastric varices
	456.8:4-5	Gastric varices
	567.2:9	Spontaneous bacterial peritonitis
	789.5	Ascites
Biliary disease	574	Cholelithiasis
	575	Disorders of gallbladder
	576	Disorders of biliary tract
Gastrointestinal hemorrhage	578	Gastrointestinal hemorrhage
**Diabetes**		
Diabetes mellitus	250	Diabetes mellitus
**Cancers**		
Malignant neoplasm	140-149	Malignant neoplasm of lip, oral cavity, and pharynx
	150-159	Malignant neoplasm of digestive organs and peritoneum
	160-165	Malignant neoplasm of respiratory and intrathoracic organ
	170-176	Malignant neoplasm of bone, connective tissue, skin, and breas
	179-189	Malignant neoplasm of genitourinary organs
	190-199	Malignant neoplasm of other and unspecified sites
	200-209	Malignant neoplasm of lymphatic and hematopoietic tissue
	235-238	Neoplasms of uncertain behavior
	239	Neoplasms of unspecified nature
Chemotherapy	V58.1	Encounter for antineoplastic chemotherapy and immunotherapy
	V66.2	Convalescence following chemotherapy
	V67.2	Follow-up examination, following chemotherapy

	99.25	Injection or infusion of cancer chemotherapeutic substance
History of cancer	V10	Personal history of cancer
**Nervous system diseases**		
Cerebrovascular events	430	Subarachnoid hemorrhage
	431	Intracerebral hemorrhage
	432	Other and unspecified intracranial hemorrhage
	433	Occlusion and stenosis of precerebral arteries
	434	Occlusion of cerebral arteries
	435	Transient cerebral ischemia
	436	Acute, but ill-defined, cerebrovascular disease
	437	Other and ill-defined cerebrovascular disease
	438	Late effects of cerebrovascular disease
Other nervous system disease	320-327	Inflammatory diseases of the central nervous system
	330-337	Hereditary and degenerative diseases of the central nervous system
	340-345	Other disorders of the central nervous system
**Respiratory diseases**		
Pneumonia	480	Viral pneumonia (other than SARS-CoV and SARS-CoV-2)
	481	Pneumococcal pneumonia [Streptococcus pneumoniae pneumonia]
	482	Other bacterial pneumonia
	483	Pneumonia due to other specified organism
	484	Pneumonia in infectious diseases classified elsewhere
	485	Bronchopneumonia, organism unspecified
	486	Pneumonia, organism unspecified
Influenza with respiratory manifestations	487	Influenza with respiratory manifestations
Chronic obstructive pulmonary disease and allied conditions	490-496	Chronic obstructive pulmonary disease and allied conditions
Pneumoconioses and other lung diseases due to external agents	500-508	Pneumoconioses and other lung diseases due to external agents
Other diseases of respiratory system	510-519	Other diseases of respiratory system
**Kidney diseases**		
Nephritis, nephrotic syndrome, and nephrosis	581	Nephrotic syndrome
	582	Chronic glomerulonephritis
	583	Nephritis and nephropathy not specified as acute or chronic
	584	Acute kidney failure
	585	Chronic kidney disease
	586	Renal failure, unspecified
	587	Renal sclerosis, unspecified
	588	Disorders resulting from impaired renal function
Renal replacement therapy	V56	Encounter for dialysis and dialysis catheter care
	38.95	Venous catheterization for renal dialysis
	39.27	Arteriovenostomy for renal dialysis
	39.42	Revision of arteriovenous shunt for renal dialysis
	39.43	Removal of arteriovenous shunt for renal dialysis
	39.95	Hemodialysis
	54.98	Peritoneal dialysis
**Human immunodeficiency virus (HIV) infection**		
HIV	042	HIV disease.
HIV	079.53	HIV, type 2 [HIV-2]
HIV	V02.9:1	HIV carrier
HIV	V08	Asymptomatic HIV infection status

ICD-9-CM = International Classification of Diseases, Ninth Revision, Clinical Modification.

Table S2. Associations of socioeconomic position, multimorbidity and other risk factors with COVID-19 severity based on multi-level binary logistic regression with random effects specified for all individual-level variables

	**Severe COVID-19**	
	aOR [95% CI] [a]	*p*-value
**Age, year**	1.04 [1.04-1.05]	<0.001
**Sex**		
Female	Ref	
Male	2.02 [1.57-2.59]	<0.001
**Presence of symptom onset**		
Asymptomatic	Ref	
Symptomatic	1.95 [1.34-2.84]	0.001
**Multimorbidity**		
Non-multimorbid	Ref	
Multimorbid	2.26 [1.71-2.98]	<0.001
**Housing type**		
Public rental housing	Ref	
Subsidized home ownership	1.00 [0.66-1.52]	0.997
Private housing	1.10 [0.82-1.48]	0.536
Residential care homes	1.39 [0.85-2.27]	0.188
Others	1.53 [0.83-2.83]	0.171
**Area-level income-poverty rate**		
Low	Ref	
Medium	1.11 [0.69-1.77]	0.669
High	1.17 [0.74-1.86]	0.503
**Area-level population density**		
Low	Ref	
Medium	1.37 [0.86-2.18]	0.182
High	1.35 [0.85-2.13]	0.203

[a] Variables listed above were mutually adjusted

Table S3. Modification of the effect of multimorbidity on COVID-19 severity by quartiles of area-level income-poverty rate

	Non-multimorbid		Multimorbid		Effect for multimorbidity within strata of area-level income-poverty rate	
	aOR [95% CI] [a]	*p*-value	aOR [95% CI] [a]	*p*-value	aOR [95% CI] [a]	*p*-value
**Area-level income-poverty rate**						
1st quartile (lowest poverty rate)	Ref		0.76 [0.22-2.57]	0.659	0.76 [0.22-2.57]	0.659
2nd quartile	0.76 [0.33-1.80]	0.538	1.23 [0.52-2.90]	0.644	1.60 [0.80-3.23]	0.187
3rd quartile	0.72 [0.33-1.58]	0.414	2.10 [0.96-4.57]	0.062	2.75 [1.53-4.91]	0.001
4th quartile (highest poverty rate)	0.80 [0.36-1.79]	0.583	2.03 [0.91-4.54]	0.085	2.65 [1.43-4.91]	0.002

Measure of effect modification of multimorbid*2nd quartile of income-poverty rate on multiplicative scale: ratio of aORs [95% CI] = 2.11 [0.52–8.49]; *p*-value = 0.293.
Measure of effect modification of multimorbid*3rd quartile of income-poverty rate on multiplicative scale: ratio of aORs [95% CI] = 3.83 [1.06–13.86]; *p*-value = 0.041.
Measure of effect modification of multimorbid*4th quartile of income-poverty rate on multiplicative scale: ratio of aORs [95% CI] = 3.35 [0.90–12.51]; *p*-value = 0.072.
Measure of effect modification of multimorbid*2nd quartile of income-poverty rate on additive scale: $RERI_{OR}$ [95% CI] = 0.73 [-0.33–1.80]; *p*-value = 0.178.
Measure of effect modification of multimorbid*3rd quartile of income-poverty rate on additive scale: $RERI_{OR}$ [95% CI] = 1.77 [0.51–3.03]; *p*-value = 0.006.
Measure of effect modification of multimorbid*4th quartile of income-poverty rate on additive scale: $RERI_{OR}$ [95% CI] = 1.57 [0.23–2.90]; *p*-value = 0.022.

[a] Adjusted for age, sex, presence of symptom onset, housing type, and area-level population density.
